# Small molecules remain on target for c-Myc

**DOI:** 10.7554/eLife.22915

**Published:** 2017-01-19

**Authors:** Linchong Sun, Ping Gao

**Affiliations:** CAS Key Laboratory of Innate Immunity and Chronic Disease, University of Science and Technology of China, Hefei, China; CAS Key Laboratory of Innate Immunity and Chronic Disease, University of Science and Technology of China, Hefei, Chinapgao2@ustc.edu.cn

**Keywords:** Reproducibility Project: Cancer Biology, replication, metascience, reproducibility, bromodomain inhibitor, myeloma, Human, Mouse

## Abstract

Targeting the transcription factor c-Myc via one of its coactivator proteins is a promising strategy for cancer therapy.

**Related research articles** Aird F, Kandela I, Mantis C, Reproducibility Project: Cancer Biology. 2017. Replication Study: BET bromodomain inhibition as a therapeutic strategy to target c-Myc. *eLife* **6**:e21253. doi: 10.7554/eLife.21253

Kandela I, Jin HY, Owen K, Reproducibility Project: Cancer Biology. 2015. Registered Report: BET bromodomain inhibition as a therapeutic strategy to target c-Myc. *eLife ***4**:e07072. doi: 10.7554/eLife.07072

Cancers develop as a result of cancerous proteins being activated or tumor suppressor proteins being inactivated. The transcription factor c-Myc is an example of a cancerous protein: this transcription factor is overexpressed in a majority of human cancers, so targeting it has long been viewed as a potential approach to cancer therapy.

c-Myc usually works in partnership with another transcription factor, called MAX, and a variety of cofactors. c-Myc and MAX form a dimer that binds to DNA and then recruits and interacts with one or more cofactors to promote the transcription of certain target genes (Lin et al., 2012; Thomas et al., 2015). However, c-Myc is also involved in many cellular processes in healthy cells, and this has complicated efforts to target it for therapeutic applications.

There are at least three reasons why it is difficult to target c-Myc and/or its cofactors (Fletcher and Prochownik, 2015). First, many cancers are caused by specific mutations, and it is possible to design small molecules that target specific mutation sites. However, in the case of c-Myc, cancer is caused by overexpression, which makes targeting more difficult. Second, we could target some of the cofactors, but we do not know how or where they bind to c-Myc. Third, like c-Myc itself, some of these cofactors also have essential roles in normal cells. However, we can exploit the fact that c-Myc binds to DNA in regions of high acetylation.

In 2011 researchers at the Dana-Farber Cancer Institute, Harvard Medical School and other institutes in the US decided to explore if it was possible to target c-Myc by inhibiting a protein called BRD4 (Delmore et al., 2011). This protein belongs to the BET (short for bromodomain and extra-terminal) subfamily of human bromodomain proteins. Delmore et al. focused on BRD4 for two reasons: it is known to bind to DNA in regions of high acetylation; and it is known to regulate gene transcription by recruiting a protein called P-TEFb, which is one of the cofactors involved in the gene transcription network regulated by c-Myc (Figure 1). They found that JQ1, a small molecule that inhibits BRD4, can inhibit the progression of certain kinds of blood cancer both in vitro and in vivo (Delmore et al., 2011).

**Figure 1.**
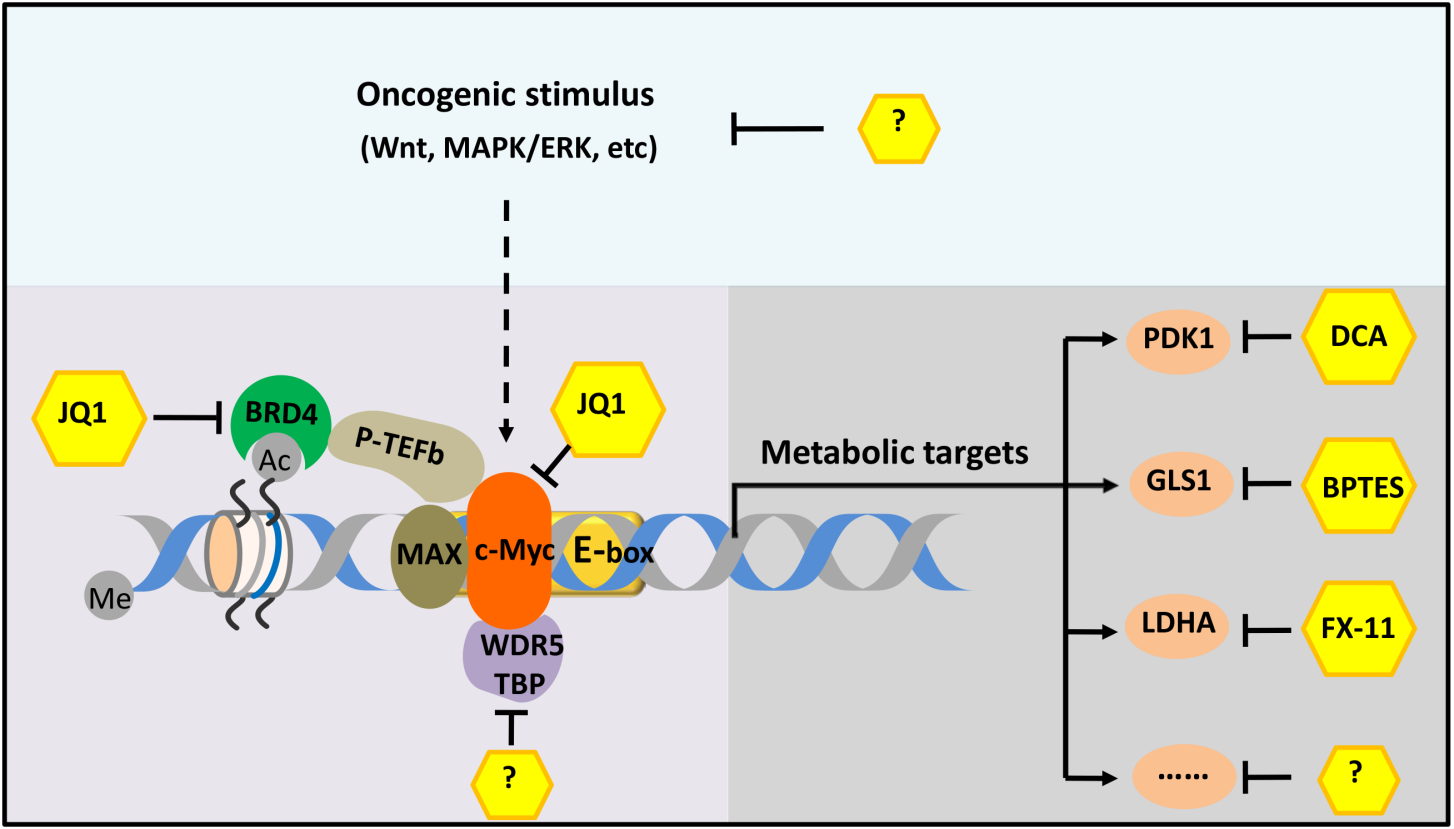
The c-Myc-related gene transcription network. The transcription factor c-Myc (orange) is a cancerous protein that is also involved in metabolism and other processes in normal cells. The small molecule JQ1 inhibits the progress of cancers in mouse models by inhibiting both c-Myc itself and one of its coactivators (BRD4; green). More effective therapy could be achieved by targeting two or more molecules or processes in the network. For example, the different pathways that lead to the cancer (such as the Wnt and MAPK/ERK pathways) could be targeted, as could various cofactors (such as WDR5 and TBP) and different metabolic enzymes (such as PDK1, GLS1 and LDHA). In some cases, such as for some of the metabolic enzymes, small-molecule inhibitors are already known; in other cases, such inhibitors have not yet been discovered (indicated by question marks). Me and Ac represent methylation and acetylation. BRD4: bromodomain-containing protein 4. P-TEFb: positive transcription elongation factor complex b.

In 2015, as part of the Reproducibility Project: Cancer Biology, Kandela et al. published a Registered Report (Kandela et al., 2015) which explained in detail how they would seek to replicate selected experiments from Delmore et al. The results of these experiments have now been published as a Replication Study (Aird et al., 2017). The Replication Study broadly confirms that JQ1 can repress the progression of certain blood cancers by targeting cofactors of c-Myc.

In the original work, Delmore et al. measured gene expression and cell proliferation in vitro (in blood cancer cell lines) before and after treatment with JQ1, and observed that treatment with JQ1 resulted in significant changes in the transcription of many (but not all) of the genes in the c-Myc-related gene transcription network: 88 genes were down-regulated by a factor of two or more, and 25 were up-regulated by a similar factor. In particular, the transcription of c-Myc was down-regulated. They also evaluated the effect of JQ1 in vivo (in a mouse model of blood cancer) and showed that treatment with JQ1 decreased the amount of c-Myc protein in the cells, reduced the proliferation of cancer cells and increased survival.

In the replication study, Aird et al. observed that JQ1 treatment down-regulated the transcription of c-Myc in vitro and increased the overall survival of the mouse model in vivo, which is consistent with the original paper. However, the decrease of tumor burden (as measured by bioluminescence) in vivo was not statistically significant, possibly because many of the control mice had to be euthanized before the pre-specified endpoint because of disease progression and high tumor burden.

Aird et al. also used an enantiomer of JQ1 that cannot inhibit BET proteins as a control. Although this enantiomer does not affect the transcription of c-Myc, it does somewhat surprisingly display pharmacological activity in mouse models (possibly caused by the enantiomer unexpectedly interacting with or metabolizing various molecules in vivo).

Other research groups have found that the inhibition of BET bromodomain proteins has suppressed the expression of c-Myc in a range of cell lines and in mouse models for other blood cancers (Dawson et al., 2011; Dawson and Kouzarides, 2012). A number of molecules that inhibit BET bromodomain proteins are also undergoing clinical trials (Berthon et al., 2016). However, treatment with JQ1 did not affect the transcription of c-Myc in other cell lines, including embryonic stem cells and certain cells in lung cancers, so certain cancers will be resistant to JQ1 treatment (Horne et al., 2015; Lockwood et al., 2012; Shimamura et al., 2013).

It is not understood why JQ1 affects some but not all of the genes in the c-Myc-related gene transcription network. Are unknown cofactors involved in the transcription, or are variations in the acetylation state important? Whatever the answer, it may be possible to combine BET bromodomain inhibitors with other small molecules for cancer therapy.

Moreover, in addition to acetylation, other forms of epigenetic modification (such as DNA methylation and nucleosome remodeling) might be important in the c-Myc-related gene transcription network. Theoretically, any of the molecules or processes in the network could be targeted to curb the oncogenic effects of c-Myc, just as BRD4 can be targeted. And since the targeting of metabolic enzymes has proved effective in mouse tumor models, it might be possible to develop new therapies based on the fact that c-Myc has a role in controlling cellular metabolism (Figure 1; Bonnet et al., 2007; Le et al., 2010; Xiang et al., 2015).

The sheer complexity of the c-Myc-related gene transcription network poses many challenges for researchers, but it also offers many opportunities for the development of more effective and specific therapies for many cancers, including human cancers that are resistant to JQ1.

## Note

Ping Gao was one of the reviewers for the Registered Report (Kandela et al., 2015) and the Replication Study (Aird et al., 2017).
